# Determinants of Infertility among Married Women Attending Dessie Referral Hospital and Dr. Misganaw Gynecology and Obstetrics Clinic, Dessie, Ethiopia

**DOI:** 10.1155/2020/1540318

**Published:** 2020-03-27

**Authors:** Desalegn Bayu, Gudina Egata, Bereket Kefale, Tadeg Jemere

**Affiliations:** ^1^Department of Public Health, College of Medicine and Health Sciences, Wollo University, Dessie, Ethiopia; ^2^Department of Public Health, College of Health Sciences, Haramaya University, Harar, Ethiopia; ^3^Department of Biomedical Sciences, College of Health Sciences, Debre Tabor University, Debre Tabor, Ethiopia

## Abstract

**Background:**

Infertility is the inability to become pregnant after one year of sexual intercourse without the use of contraception. Epidemiological data suggest that 10 to 15% of couples around the world are suffering from infertility. The exact meaning of marriage is mainly fulfilled if the couple conceives and bears children. Failure of this often leads to unhappy married lives, divorces, and high levels of psychiatric morbidity. There is scarcity of data about determinants of infertility in Ethiopia. Therefore, the aim of this study is to identify the determinants of infertility among married women attending Dessie Referral Hospital and Dr. Misganaw gynecology and obstetrics specialty clinic, Dessie, Ethiopia, 2019.

**Methods:**

An institution-based case-control study was conducted on 281 participants. The participants were selected by a systematic random sampling technique. Data were collected using a structured interviewer-administered questionnaire. Data were entered into EpiData version 3.1 and exported to SPSS version 23 for analyses. Variables with *p* < 0.25 in bivariable logistic regression were entered into multivariable logistic regression. Odds ratio with its 95% confidence interval was estimated to measure the direction and strength of the association. The level of statistical significance was set at *p* < 0.05.

**Results:**

The determinants of women's infertility were age at the first pregnancy (AOR = 2.89; 95% CI: 1.105, 7.564), age at menarche (AOR = 3.2; 95% CI: 1.278, 7.975), menstruation flow in days (AOR = 4.17; 95% CI: 0.062, 0.929), multiple sexual partners (AOR = 5.33; 95% CI: 2.124, 13.397), and history of STI (AOR = 2.79; 95% CI: 1.088, 7.159).

**Conclusion:**

Age at the first pregnancy, age at menarche, multiple sexual partners, number of days of menstruation flow, and history of STI were determinants of women's infertility. Infertility may bring about unhappy married lives, divorces, and high levels of psychiatric morbidity. Therefore, couples need to have heath education about risk factors for infertility.

## 1. Introduction

Infertility is a disease of the reproductive system characterized by the failure to achieve pregnancy for 12 months or more with regular unprotected sexual intercourse, or it is the inability of a sexually active, noncontraceptive couple to achieve pregnancy in one year [1–3]. It can be classified as primary and secondary. Primary infertility is termed as if conception has never occurred, whereas secondary infertility means that the patient fails to conceive after having achieved a previous conception [4]. Infertility in a couple can be due to problems in either women or men, not necessarily both. It was found that 1/3^rd^ of the time fertility problems lie with the man, 1/3^rd^ of the time with women, and 1/3^rd^ of the time with both men and women [5].

The result of different studies showed that 10 to 15% of couples around the world are suffering from infertility [1, 6], and the 2012 World Health Organization (WHO) report indicated that the general burden of female infertility has continued to be the same in the estimated levels and trends from 1990 to 2010 [7].

The exact meaning of marriage is mainly fulfilled if the couple conceives and bears children. Failure of this often leads to unhappy married lives, divorces, and high levels of psychiatric morbidity [8]. Different studies have also shown that couples with infertility often face social stigmatization and are publically isolated [9]. Domestic violence and dissolution of marriage occur significantly more often among the infertile couples compared with couples in fecund relationships [8].

According to the findings of different studies, reproductive system disorders like sexually transmitted disease and endometrial and ovarian dysfunction, genetic factor, age, alcohol consumption, smoking, obesity, irregular menstrual cycle, age at menarche, age at marriage, and dysmenorrhea are found to be factors affecting infertility [3, 10, 11].

In Ethiopia, no study has comprehensively evaluated the burden and determinant factors of infertility, even though it is well documented in developed countries [1, 6]. Hence, the main aim of this study is to identify the determinants of infertility among married women.

## 2. Methods

### 2.1. Study Design, Area, and Period

An institution-based unmatched case-control study was conducted at Dessie Referral Hospital (DRH) and Dr. Misganaw gynecology and obstetrics specialty clinic, in Dessie town from January 3 to March 8, 2019. Dessie town is located in the Amhara regional state, 401 kilometers from northeast of Addis Ababa. Dessie Referral Hospital is one of the oldest hospitals in Ethiopia, providing services for approximately 3 million people in the catchment area including infertility.

### 2.2. Source and Study Population

All infertile married/cohabited women aged 15 to 49 years and all married/cohabited women aged 15 to 49 years who are on the first postnatal care at DRH and Dr. Misganaw gynecology and obstetrics specialty clinic were source populations. The sample of infertile married/cohabited women aged 15 to 49 years and the sample of married/cohabited women aged 15 to 49 years who are on the first postnatal care at DRH and Dr. Misganaw gynecology and obstetrics specialty clinic present at the time of data collection were the study populations.

### 2.3. Eligibility Criteria

The women who fail to achieve a clinical pregnancy for 12 months or more with regular unprotected sexual intercourse and married/cohabited women aged 15 to 49 years who are on the first postnatal care at DRH and Dr. Misganaw gynecology and obstetrics specialty clinic present at the time of data collection were included. Women whose male partners are infertile and who have hearing and speaking problem were excluded.

### 2.4. Sample Size and Sampling Technique

The required sample size was computed using open Epi Info version 7.2.1 by considering the assumption that the ratio of cases to controls is 1 : 2, the power is 90, the confidence level is 95%, the odds ratio (OR) is 3.632, the proportion of exposed cases (P_1_) is 24, and the proportion of exposed controls (P_2_) is 8 (by taking the duration of the menstruation cycle associated with female infertility from the recent study conducted in India) [5]. Taking the response rate of 10%, the final sample size was 291, and since the ratio of cases to controls was 1 : 2, 97 samples were cases and 194 were taken as controls. Study participants were selected using a systematic random sampling technique for both cases and controls.

### 2.5. Operational Definitions

*Cases* are married/cohabited women aged between 15 and 49 years who failed to achieve a clinical pregnancy after 12 months or more with regular unprotected sexual intercourse.

*Controls* are married/cohabited women aged between 15 and 49 years who are on the first postnatal care at Dessie Referral Hospital and Dr. Misganaw gynecology and obstetrics specialty clinic.

*Normal male sperm analysis* is when all parameters are in normal range (the volume > 1.5 ml, sperm concentration > 15 million sperm/ml, shape > 4%, and motility > 32% with forward progression) [12].

*First postnatal care* is the health care for women and their child for the first 24 hours after the delivery was completed.

*History of contraceptive use* is the use a contraceptive method (COC, IUD, implants, and injectable) before 2 years.

*Uterine abnormality* is the female uterus that differs from the normal structure and position of the uterus [13].

*Substance use* is an intentional ingestion of one or more psychostimulant drugs (alcohol, khat, and cigarette smoking). Individuals who use any of the substances at least once in their lifetime were classified as ever users, and those who consumed at least once within the last 30 days were classified as current users.

### 2.6. Data Collection Procedure

Data were collected using a pretested interviewer-administered structured questionnaire. Weight and height were measured using a digital weight scale and a stadiometer, respectively. Body Mass Index (BMI) was calculated for each participant.

Medical and laboratory results were taken from the participant's medical folder. The women were asked during their visit to health facilities for health services. Data were collected by four trained BSc nurses.

### 2.7. Data Analysis Procedure

Data were checked for completeness and entered into EpiData version 3.1 and exported to SPSS version 23 (IBM, New York, USA) for analyses. Bivariable logistic regression analyses were done to see the association between each independent variable and the outcome variable. Variables with *p* < 0.25 were entered into backward stepwise multivariable logistic regression to control for all possible confounders and to identify determinants of infertility. We used *p* < 0.25 as a cutoff point to select candidate variables of the final model so as to improve the chances of retaining meaningful confounders. Odds ratio with its 95% confidence interval was estimated to identify the determinants of infertility. The level of statistical significance was set at *p* < 0.05.

### 2.8. Data Quality Management

Emphasis was given for developing the data collection instrument. Training for data collectors was given for 2 days regarding the purpose of the study, interview, and measurement techniques. Questionnaires were translated into the Amharic language and then retranslated back to English for its consistency. A week before the actual time of data collection, a pretest was done on 5% of the participants having similar sociocultural characteristics with the study participants at Selam General Hospital.

## 3. Results

### 3.1. Sociodemographic Characteristics of the Study Participants

A total of 281 participants (93 cases and 188 controls) were included in the study having a response rate of 96.6%. The mean age of cases and controls was 29.02 (±6.38) and 29.59 (±6.9) years, respectively. The mean age at marriage was 19.77 (±3.83) years for cases and 19.22 (±3.71) years for controls. Fifty-three (57%) of cases and 108 (57.4%) of controls lived in the urban area. Sixty-seven (72%) of cases and 168 (89.4%) of controls had no family history of infertility (Table 1).

### 3.2. Obstetric History of Study Participants

Eighty (86%) of cases and 134 (71.3%) of controls had greater than 3-day menstruation flow. Twenty-six (28%) of cases and nearly half (93 (49.5%)) of controls had a history of the first pregnancy before 21 year. Forty-eight (51.6%) of cases and 75 (39.9%) of controls had a history of dysmenorrhea. Forty-eight (51.6%) of cases and 91 (48.4%) of controls had an irregular menstrual pattern. Forty (43%) of cases and 63 (33.5%) of controls had uterine abnormality. Sixty-three (67.7%) of cases and 75 (39.9%) of controls started their first menstruation at the age of 14 and above.

### 3.3. Lifestyle and Behavioral Characteristics of Study Participants

Eighteen (19.4%) of cases and 32 (17%) of controls had a history of cigarette smoking. The lifetime prevalence of khat chewing was 22.6% among cases and 18.6% among controls. The lifetime prevalence of alcohol drinking was 21.5% among cases and 18.1% among controls. Twenty-one (22.6%) of cases and 33 (17.6%) of controls were found to be underweight. On the other hand, 43 (46.2%) of cases and 75 (39.7%) of controls were overweight. Thirteen (14%) of cases and 43 (22.9%) of controls were found to be obese.

### 3.4. Sexual Characteristics of Study Participants

From the total respondents, 37 (39.8%) of cases and 35 (18.6%) of controls had multiple sexual partners. Regarding history of STI, 43 (46.2%) of cases and 30 (16%) of controls had a history of sexually transmitted infection and the current proportion of women with STI was 33.3% for cases and 31.4% for controls. On the other hand, 18.3% of cases and 15.4% of controls were positive for HIV.

### 3.5. Family Planning Characteristics of Study Participants

Among the total participants, 38 (40.9%) of cases and 82 (43.6%) of controls used family planning before two years. Seventeen (44.7%) of cases and 39 (20.7%) of controls used injectable family planning devices, whereas fourteen (15%) of cases and 26 (13.8%) of controls used implants. On the other hand, 7 (18.4%) of cases and 17 (9%) of controls use combined oral contraceptive.

### 3.6. Determinants of Women's Infertility

In the multivariable regression model, age at the first pregnancy (AOR = 2.89; 95% CI: 1.105, 7.564), age at menarche (AOR = 3.2; 95% CI: 1.278, 7.975), menstruation flow in days (AOR = 4.17; 95% CI: 0.062, 0.929), multiple sexual partners (AOR = 5.33; 95% CI: 2.124, 13.397), and history of STI (AOR = 2.79; 95% CI: 1.088, 7.159) were significantly associated with women's infertility.

The odds of infertility among women whose age at the first pregnancy is less than 21 years were 2.9 times higher than those among women whose age at the first pregnancy is greater than or equal to 21 years. Similarly, the odds of infertility among women whose age at the first menstruation is greater than or equal to 14 years were 3.2 times higher than those among women whose age at the first menstruation is less than 14 years. The odds of infertility among women whose duration of menses was greater than 3 days were 4.2 times higher than those among women whose duration of menses was less than or equal to 3 days. Women who had multiple sexual partners had 5.3 times more chance of infertility than those who did not have multiple sexual partners. Women who had a history of STI were 2.8 times more likely to be infertile than those who did not have a history of STI (Table 2).

## 4. Discussion

This study attempted to assess determinants of infertility among women who were seeking health service in Dessie Referral Hospital and Dr. Misganaw gynecology and obstetrics specialty clinic in Dessie town. Accordingly, variables like age at the first pregnancy, age at menarche, multiple sexual partners, history of STI, and menstruation flow were identified as determinants of women's infertility. Our findings were in line with the studies conducted in Brazil [14], India [5], Rwanda [4], Nigeria [15], and Tanzania [16]. This might be due to the similarity in socioeconomic and awareness level of populations in these countries.

The odds of infertility among women whose age at the first pregnancy was less than 21 years were 2.9 times higher than those among women whose age at the first pregnancy was greater than or equal to 21 years controlling all other factors in the model. This is comparable with the studies conducted in Rwanda [4] and India [5]. This might be due to the fact that women under 21 are not mentally and physically matured for pregnancy and delivery and it may affect their uterus as well as hormonal status like estrogen and progesterone [14].

Moreover, the odds of infertility among women whose age at menarche was greater than or equal to 14 years were 3.2 times higher than those among women whose age at menarche was less than 14 years. This result is in line with a study conducted in Brazil [14]. Menarche can be delayed by genetic factors, hypothalamus-pituitary-ovarian axis abnormalities, undernutrition, hard physical exercise, psychological factors, or chronic disease. These factors affect not only the menarche but also subsequent fertility of the women [17, 18].

Another significant determinant in this study was menstruation flow. The odds of infertility among women whose menstruation flow was greater than three days were 4.2 times higher than those among women whose menstruation flow was less than or equal to three days. This result is similar to the result of the studies conducted in India [5] and China [19]. This might be related to hormonal disorders. The inability of women at ovulation and regulation of hormone levels leads to hormonal imbalances. These hormonal disorders are characterized by symptoms such as irregular menstrual cycles, excessive bleeding or very little bleeding, absence of menstruation, or long menstruation periods which are risk factors for infertility [3].

This study also showed that women who had multiple sexual partners had 5.3 times more chance of infertility than those who did not have multiple sexual partners. This result is similar to the studies conducted in Tanzania [16] and Cameroon [20]. This might be due to the fact that females with multiple sexual partners are at higher risk of acquiring STI, which by itself is a risk factor for infertility.

Women who had a history of STI were 2.8 times more likely to be infertile than those who did not have a history of STI. This result is in line with the studies conducted in Tanzania [16] and Nigeria [15]. The possible justification could be due to the fact that women with a history of STI are more likely to become infertile because chlamydial and gonococcal infections like salpingitis and pelvic inflammatory disease cause alterations in the tubal mucosa, intratubal adhesion, and distal tubal obstruction which finally result in infertility by interfering fertilization [21]. According to the study conducted in the USA, approximately 15% of women with PID develop infertility, and the number of episodes of PID a woman experiences is directly proportional to her risk of infertility [22].

## 5. Limitations of the Study

This study could have the following limitation: it did not assess endocrine factors (thyroid hormone and lactation hormones).

## 6. Conclusion

Age at the first pregnancy, age at menarche, multiple sexual partners, number of days of menstruation flow, and history of STI were significant determinants of female infertility.

## Figures and Tables

**Table 1:** Sociodemographic characteristics of study participants at DRH and Dr. Misganaw gynecology and obstetrics specialty clinic, Dessie, Ethiopia, 2019.

Variable	Category	Case, *n* (%)	Control, *n* (%)
Age	≤30	61 (65.6%)	109 (58%)
	>30	32 (34.4%)	79 (42%)
Residence	Urban	53 (57%)	108 (57.4%)
	Rural	40 (43%)	80 (42.6%)
Religion	Orthodox Christian	30 (32.3%)	84 (44.7%)
	Muslim	40 (43%)	58 (30.9%)
	Catholic	11 (11.8%)	22 (11.7%)
	Protestant	12 (12.9%)	24 (12.8%)
Region	Amhara	56 (60.2%)	96 (51.1%)
	Tigray	18 (19.4%)	49 (26.1%)
	Afar	19 (20.4%)	43 (22.9%)
Education	Illiterate	33 (35.5%)	72 (39.4%)
	Primary education	29 (31.2%)	58 (30.9%)
	Secondary and preparatory education	15 (16.1%)	27 (14.4%)
	College and above	16 (17.2%)	29 (15.4%)
Occupation	Merchant	19 (20.4%)	50 (26.6%)
	Daily labor	22 (23.7%)	51 (27.1%)
	Housewife	37 (39.8%)	56 (29.8%)
	Government/NGO employee	15 (16.1%)	31 (16.5%)
Husband place	With you	72 (77.4%)	140 (74.5%)
	Without you	21 (22.6%)	48 (25.5%)
Age at marriage	<20	46 (49.5%)	98 (52.1%)
	≥20	47 (50.5%)	90 (47.9%)
Family history of infertility	Yes	26 (28%)	20 (10.6%)
	No	67 (72%)	168 (89.4%)

**Table 2:** Determinants of infertility among women seeking heath service at DRH and Dr. Misganaw gynecology and obstetrics specialty clinic, Dessie, Ethiopia, 2019.

Variable	Category	Case, *n* (%)	Control, *n* (%)	COR (95% CI)	AOR (95% CI)
Age	≤30	61 (65.6%)	109 (58%)	1.38 (0.824, 2.316)	0.85 (0.332, 2.188)
	>30	32 (34.4%)	79 (42%)	1	1
Family history of infertility	Yes	26 (28%)	20 (10.6%)	3.26 (1.705, 6.232)	1.88 (0.577, 6.134)
	No	67 (72%)	168 (89.4%)	1	1
Type of pregnancy	Wanted	20 (58.8%)	131 (69.7%)	1	1
	Unwanted	14 (41.2%)	57 (30.3%)	1.61 (0.760, 3.407)	0.72 (0.235, 2.195)
Outcome of pregnancy	Normal	11 (32.4%)	122 (64.9%)	1	1
	Abortion	16 (47.1%)	45 (23.9%)	3.94 (1.702, 9.138)	2.22 (0.814, 6.066)
	Stillbirth	7 (20.6%)	21 (11.2%)	3.7 (1.288, 10.615)	2.89 (0.747, 11.14)
Age of first pregnancy	<21	26 (76.5%)	93 (49.5%)	3.32 (1.430, 7.709)	2.89 (1.105, 7.564)^∗∗^
	≥21	8 (23.5%)	95 (50.5%)	1	1
Dysmenorrhea	Yes	48 (51.6%)	75 (39.9%)	1.61 (0.974, 2.651)	0.52 (0.200, 1.368)
	No	45 (48.4%)	113 (60.1%)	1	1
Menstruation flow in days	≤3	13 (14%)	54 (28.7%)	1	1
	>3	80 (86%)	134 (71.3%)	2.48 (1.274, 4.826)	4.17 (1.077, 16.178)^∗∗^
Age at the first menstruation	<14	30 (32.3%)	113 (60.1%)	1	1
	≥14	63 (67.7%)	75 (39.9%)	3.16 (1.874, 5.342)	3.19 (1.278, 7.975)^∗^
Uterine abnormality	Yes	40 (43%)	63 (33.5%)	1.5 (0.899, 2.494)	0.98 (0.363, 2.624)
	No	53 (57%)	125 (66.5%)	1	1
BMI	<25	64 (68.8%)	108 (57.4%)	1	1
	≥25	29 (31.2%)	80 (42.6%)	0.61 (0.362, 1.035)	0.66 (0.246, 1.746)
Multiple sexual partners	Yes	37 (39.8%)	35 (18.6%)	2.89 (1.659, 5.028)	5.34 (2.124, 13.397)^∗^
	No	56 (60.2%)	153 (81.4%)	1	1
History of STI	Yes	28 (75.7%)	30 (85.7%)	4.53 (2.576, 7.964)	2.79 (1.088, 7.159)^∗^
	No	43 (46.2%)	30 (16%)	1	1

COR = crude odds ratio; AOR = adjusted odds ratio; CI = confidence interval. ^∗^*p* < 0.001, ^∗∗^*p* < 0.05.

## Data Availability

The authors confirm that all data underlying the findings are fully available without restriction. All relevant data are within the manuscript.
